# Testing a Conceptual Model of Early Adversity, Neural Function, and Psychopathology: Protocol for a Retrospective Observational Cohort Study

**DOI:** 10.2196/59636

**Published:** 2024-09-17

**Authors:** Nicolas Murgueitio, Maresa Tate, Lucy Lurie, Zoe Priddy, Sneha Boda, Michelle Shipkova, Micaela Rodriguez, Laura Machlin, Sarah Furlong, Amanda Mitchell, Katie McLaughlin, Margaret Sheridan

**Affiliations:** 1 Department of Psychology and Neuroscience, The University of North Carolina at Chapel Hill Chapel Hill, NC United States; 2 The Ballmer Institute for Children’s Behavioral Health, University of Oregon Eugene, OR United States

**Keywords:** early adversity, psychopathology, neurodevelopment, adverse childhood events, child development

## Abstract

**Background:**

Early adversity, broadly defined as a set of negative exposures during childhood, is extremely common and increases risk for psychopathology across the life span. Previous research suggests that separate dimensions of adversity increase risk through developmental plasticity mechanisms shaping unique neurobiological pathways. Specifically, research suggests that deprivation is associated with deficits in higher order cognition, while threat is associated with atypicality in fear learning and emotion dysregulation. However, most of this research has been conducted in adolescent and adult samples, long after exposure to adversity occurs and far from periods of peak developmental plasticity.

**Objective:**

The Wellness Health and Life Experiences (WHALE) study examines the neurobiological and behavioral mechanisms by which deprivation, threat, and unpredictability increase risk for psychopathology in early childhood (age 4-7 years) directly following periods of peak developmental plasticity. The objective of this study is to describe the study rationale and aims, the research design and procedures, and the analytical plan to test the study hypotheses.

**Methods:**

This is a retrospective cohort study that examines associations between exposure to deprivation and threat and their hypothesized neurobiological mechanisms, how these neurobiological mechanisms link early adversity and psychopathology, and associations between unpredictability, reward learning, and psychopathology. The sample was a convenience sample of children (aged 4-7 years) and their families, identified through flyers, email blasts to listserves, school-based advertising, and involvement in community events. Data were collected during a home visit, a subsequent laboratory visit, and a final neuroimaging visit. Planned analyses include linear regression, path analyses, and functional magnetic resonance imaging analyses to explore the role of neural function in the association between early adversity and psychopathology.

**Results:**

Participants (N=301) have been recruited into the study, and data collection has commenced. The expected results will be available in 2024.

**Conclusions:**

The findings of this study will help elucidate the neurobiological mechanisms by which early adversity increases risk for psychopathology in early childhood. This study represents the earliest test of an influential theory of biological embedding of early adversity.

**International Registered Report Identifier (IRRID):**

DERR1-10.2196/59636

## Introduction

### Background

Exposure to early adversity (EA) is extremely common in the population, with one-half of children in the United States experiencing some form of EA [1-3]. Moreover, EA profoundly increases risk for novel onset of internalizing and externalizing psychopathology [1,2], making it a central public health concern. Childhood psychopathology is associated with increased risk for psychopathology later in life [4], and these disorders cause economic burden to public health systems (eg, estimated annual costs of US $43.2 billion) [5]. However, current efforts in this direction are stymied by a lack of understanding of the pathways through which EA increases risk for psychopathology [6].

Progress in identifying these pathways has been limited by several critical barriers, including a lack of focus on neurodevelopmental mechanisms linking EA exposure to psychopathology. Understanding neurodevelopmental mechanisms is of import because psychopathology is an equifinal outcome for numerous forms of adversity, whereas neurobiology is likely to be more specifically impacted by different forms of EA. Second, to date, most work examining the impact of adversity on neurobiology has been conducted in adolescent or adult samples, long after EA exposure and often after the onset of psychopathology. This focus on adolescents and adults limits how research findings can be used for early preventive interventions and confounds psychopathology with adversity exposure in identifying neurobiological pathways. In the study presented here, we focus on neural mechanisms linking EA to symptoms of psychopathology in early childhood.

### Aims and Hypotheses of the Wellness Health and Life Experiences Study

The primary aim of this study is to differentiate pathways linking distinct dimensions of EA to psychopathology by testing a novel conceptual framework: the dimensional model of adversity and psychopathology (DMAP) [2]. The DMAP draws on basic neuroscience principles from animal models, existing developmental data, and preliminary findings from our own laboratory [2-5,7,8]. This framework differentiates between deprivation (absence of expected cognitive learning experiences) and threat (presence of atypical traumatic learning experiences) as distinct dimensions of EA and makes predictions about their distinct effects on structural and functional brain development. The DMAP proposes that experiences of deprivation primarily influence the development of cognitive control systems, including the structure and function of the frontoparietal network, while experiences of threat primarily influence the development of negative valence systems, including the structure and function of the amygdala and medial prefrontal cortex. In this study, we additionally propose testing the impact of unpredictability (inconsistent caregiver response) on neurodevelopment and psychopathology. We expect unpredictability to have distinct and specific effects on positive valence systems, including ventral striatal function and reward learning. This study stands to contribute meaningfully to our understanding of how specific dimensions of environmental experience influence neural development to increase risk for psychopathology, addressing objectives 1 and 2 of the National Institute of Mental Health Strategic Plan.

Before this application, earlier work focused on linking single types of adversity, such as poverty, abuse, and neglect, to specific mechanistic pathways. These models of individual adversities ignore clustering of risk exposure. Associations of one form of adversity with an outcome without measuring other forms of adversity may lead to misidentification of risk pathways [9-11]. Other prior approaches used a cumulative risk approach where associations between the number of adversities of any kind with developmental outcomes were measured [1-3]. In this model all forms of adversity are grouped together and considered to have similar, equal, and additive effects on development. The most commonly proposed neurobiological mechanism for the cumulative risk model is allostatic load and disruptions in physiological stress response systems [12]. Proposing a single stress pathway is problematic as it ignores numerous other developmental mechanisms through which early experience shapes brain development. For example, poverty is associated with a broad range of child development outcomes, not only because of alterations to stress physiology but also because of differences in access to formal and informal learning opportunities (eg, access to educational resources and variety of experiences) [13]. The proposed research moves beyond the approach of collapsing across diverse experiences of adversity to one that aims to distill these complex exposures into their core underlying dimensions of experience [6]. Exposure to deprivation and threat often co-occur; however, we have accumulating evidence from our laboratories and independent replication by others [14], indicating that it is possible to observe independent effects of deprivation and threat. Importantly, we do not expect these to be the only 2 dimensions of adversity. In this proposal we include a third dimension: unpredictability.

The Wellness Health and Life Experiences (WHALE) study addresses 3 specific aims. Aim 1 addresses the selective associations between deprivation, threat, cognitive control, and fear learning. Two sets of hypotheses are proposed for aim 1. The first is that experiences of deprivation (lack of cognitive enrichment and decreased complexity of linguistic input) will be negatively associated with cognitive control, reduced cortical thickness in lateral prefrontal cortex, and reduced activation in lateral prefrontal cortex during executive function. Second, threat (experiences of interpersonal violence) will be associated with altered fear acquisition and extinction learning, reduced medial prefrontal cortex thickness, and heightened amygdala activation during emotion regulation. Aim 2 examines the distinct neurobiological pathways through which deprivation and threat predict psychopathology. A total of 2 sets of hypotheses are proposed for aim 2. We hypothesize that alterations to cognitive control and frontoparietal network structure and function will mediate the association of early childhood deprivation and psychopathology across internalizing and externalizing dimensions. In addition, we predict that altered fear learning and altered structure and function of the amygdala and medial prefrontal cortex will mediate the association of threat with psychopathology. Finally, aim 3, an exploratory aim, assesses the association of unpredictability with altered associative learning of reward and psychopathology. We hypothesize that decreased predictability of the family environment (inconsistent and low contingency of caregiver responses, instability of the family and physical environment, and family chaos) will predict blunted reward learning and ventral striatal activation. Finally, we predict that disrupted reward learning and underlying neural circuitry will mediate the association of unpredictability with psychopathology.

## Methods

### Sample

#### Power Analyses

We used functional magnetic resonance imaging (fMRI)–based power analysis implemented in the fMRIpower software package 2007 version [15] in MATLAB [16]. Power calculations were for activation in the medial prefrontal cortex (equivalent to a region of interest analysis with *P*<.05) extracted from a study conducted in our laboratory of a cognitive control task that included children as young as 5 years to estimate the size of our effect. This analysis indicated that a sample of n=45 would achieve 80% power to detect an expected medium effect size of 0.45 in the medial prefrontal cortex. Our total projected sample was >200 participants, which achieves power of >85% to detect a medium effect.

Power for the mediation analyses was examined using Monte Carlo simulations in MPlus 6.1 [17-19]. Data were simulated for a population based on estimated parameters, and a model was estimated for multiple samples drawn from that population. Power was derived based on the proportion of replications in which the null hypothesis (ie, that a given parameter is 0) is correctly rejected when it is false. We estimated power in 1000 replications to detect effects based on our estimated sample size of 200 using minimum detectable effect sizes. For path models predicting indirect effects of threat and deprivation on psychopathology via neural function (aim 2), we will have power (with a sample of 200) to detect indirect effects as small as a×b=0.067.

#### Recruitment Strategy

The sample was a convenience sample identified through flyers, email blasts to listserves, school-based advertising, and involvement in community events. The first 20 participating families were recruited into the study without regards to subsequent selection criteria and were only required to live within 1 hour of Chapel Hill, North Carolina, and to have a child aged between 4 and 7 years. Following these early participants, interested families who provided their contact information were called and a phone screen was conducted. This phone screen identified participants likely to experience EA based on identity variables related to structural inequality, self-disclosed family violence, or self-disclosed previous involvement with child protective services. Extensive prior research suggests that structural inequality related to race and class increases risk of experiencing childhood adversities, including deprivation and threat [20,21]. The screening questionnaire first asked if either caregiver had less than a high-school education or had a minoritized ethnic or racial identity. If caregivers answered positively, they were recruited into the study, if not, they answered a short series of questions about family violence or child protective services involvement. If they reported either involvement in child protective services or family violence exposure, they were recruited into the study. If they did not endorse low caregiver education, minoritized identity, family violence, or child protective services involvement, they were not recruited into the study. Prospective participants who did not meet inclusion criteria completed screening questionnaires only. Due to the “funnel” nature of the screen, not all participants were asked all screen questions (ie, once the family “screened in,” the questionnaire was terminated). Consequently, all information about family violence in the study is from subsequent study visits and not the screener form. Our recruitment strategy resulted in a diverse sample with respect to race, ethnicity, and caregiver education, and is enriched for exposure to EA, including interpersonal violence and neglect. Before participation in any study visit, some participants were screened out if the primary caregiver and child did not endorse speaking and reading English well enough to provide informed consent. Additional exclusion criteria included major medical conditions which would compromise study involvement, neurological illness, pervasive developmental disorders, or substantial prenatal substance exposure. Child participants were not included in the magnetic resonance imaging (MRI) visit if their caregiver reported the presence of MRI contraindications or history of concussion. Given the extensive nature of these criteria, some were ascertained after inclusion in initial study visits. Our final cohort (N=301) includes every caregiver participant who consented to study participation following initial phone screen regardless of if they met subsequent exclusion criteria or refused to participate following consent.

### Design and Procedures

#### Study Overview

The study consisted of 3 visits. A total of 275 (91.4%) of 301 participants participated in at least 1 study visit, 234 (77.7%) of 301 participated in 2 study visits, and 217 (72.1%) of 301 participated in 3 study visits. The same primary caregiver participated in all 3 visits, except in cases where legal custody changed between visits. Between visit 1 and 2, there was on average 3.63 (SD 4.66) months; between visit 2 and visit 3, there was on average 4.41 (SD 4.87) months. At each visit, 1 research assistant (RA) worked with the primary caregiver (caregiver RA) and another with the child (child RA). See Multimedia Appendix 1 for an overview of every measure and task included in the study.

#### Visit 1

Visit 1 took place in the participant’s home or current place of residence and lasted approximately 2.5 hours. We began with a tour of the family’s home with careful attention to the home and neighborhood environment. The child RA obtained verbal assent from the child, after which the research staff conducted interviews and tasks with the family. The caregiver RA completed interviews assessing the child’s MRI contraindications and exposure to enriched learning experiences with the child’s primary caregiver. Next, caregivers completed web-based questionnaires using Qualtrics, which assessed developmental milestones, prenatal exposure to substances, sleep hygiene, life events, and collateral contacts for longitudinal follow-up. Finally, the caregiver RA took 2 photos each of the primary caregiver and a secondary caregiver for task stimuli at visits 2 and 3. These photos were headshots taken against a neutral background in which caregivers were asked to make neutral and smiling faces. The caregiver RA compensated the family and scheduled the second study visit. The child RA began by giving the child a prize bag and building rapport. The child RA administered a series of behavioral tasks assessing executive function and reward learning to the child. For every game the child played, they received a prize, and all children were given their prizes regardless of their performance. In most cases, the caregivers and children completed their tasks in separate spaces from each other. When not possible to administer study tasks in separate spaces for caregivers and children, caregivers were instructed to not interfere with their children’s task performance.

#### Visit 2

Visit 2 was conducted in laboratory facilities on the University of North Carolina campus and was 6 hours long, including a half hour lunch break. Upon the family’s arrival, the child RA completed verbal assent with the child in their caregiver’s presence. The caregiver RA began the day with a general overview and reminders about confidentiality, and then completed interviews and surveys with caregivers. During visit 2, caregivers completed questionnaires about child behavior, symptoms of psychopathology, family functioning, and discipline practices in the home. Meanwhile, the child RA started the day with an introduction to prizes and built rapport with the child. Children completed tasks that assessed executive function, fear learning, and reward learning. They additionally completed an electroencephalogram (EEG) recording. EEG recording was accomplished using a 128-channel Sensor Net system. The net is composed of an elastic tension structure forming a geodesic tessellation of the head surface containing carbon fiber electrodes embedded in pedestal sponges. At each vertex, there is a sensor pedestal housing a silver/silver chloride–coated, carbon-filled plastic electrode and sponge containing saline electrolyte. Before fitting the Sensor Net over the scalp, the sponges are soaked in electrolyte solution (6 mL KCL per liter of distilled water) to facilitate electrical contact between the scalp and the relevant electrode. The data were amplified, filtered (bandpass 0.1-100.0 Hz), and sampled at an effective rate of 250 Hz. Before recording, measurements of channel gains and zeros were taken to provide an accurate scaling factor for display of waveform data and so that baseline correction can be performed. The participant’s head was measured and marked to ensure accurate placement, and the net is then placed over the scalp. All RAs collecting EEG data were trained by the study’s principal investigator (MS), an expert on EEG data collection and analysis. In the middle of the visit, the caregiver and child were reunited to complete video-recorded parent-child interaction (PCI) tasks [22]. The PCI tasks included 3 interactive tasks, lasting 10 minutes each, during which the caregiver and child played with toys, completed puzzles, and read a book. The child RA provided verbal instructions before each task. Families were permitted to speak in the primary language spoken at home across each task. After this, families were typically given their lunch break for 30 minutes.

After lunch, the caregiver RA and the child RA instructed the child to refrain from drinking or eating to prepare for saliva collection. The child participant completed a resting-state and task-based EEG recordings. Next, the child RA completed a 3-part saliva collection; 1 sample was taken immediately after EEG. Next, children participated in a finger prick task where a single finger prick was performed to obtain up to 5 blood spots. Children participated with their caregiver and an RA they had not previously interacted with, as these semistructured tasks were developed to measure how parents support children’s emotion regulation (see below), and we wanted to avoid children using the child RA as support. Saliva was collected before the finger prick task, 20 minutes after finger prick, and 40 minutes afterward. Finally, a mock scan was completed to prepare the child for visit 3.

#### Visit 3

Visit 3 of the WHALE study was conducted at the University of North Carolina Biomedical Research Imaging Center in Chapel Hill, United States. This visit included an MRI comprising structural (ie, magnetization-prepared rapid gradient echo [MPRAGE]), diffusion tensor imaging, and functional sequences on a 3T Siemens Prisma scanner. Upon arrival, the child RA obtained verbal assent from the child in their caregiver’s presence. Children underwent training for 3 tasks that they would later complete in the scanner, including an executive function task, a reward anticipation and receipt task, and an emotion regulation task. Families were reminded of MRI procedures. At the visit, caregivers completed a series of questionnaires assessing their own and their child’s emotion regulation, executive function, and the caregivers’ history of exposure to adversity. While being trained, the child selected prizes that they would win for completing these tasks during the MRI scan to serve as motivation. After task training, both RAs accompanied the caregiver and child to the MRI suite.

At the MRI suite, the caregiver helped the child change into MRI-safe scrubs, after which the MRI technician reviewed the completed MRI safety screening form with the caregiver. The child and caregiver were separately asked about potential MRI-unsafe materials inside the child’s body. During MRI scan, children were given earplugs and noise-canceling headphones, cushions around their head, a sheet, and a weighted blanket to ensure comfort during the scan and limit movement. The child RA stayed with the child in the scan room throughout the scan to communicate with them and respond to any child concerns. After completing MPRAGE and 1 task, each child came out of the scanner for a break and a snack. We used AAScout for slice positioning to ensure that children can get in and out of the scanner multiple times, allowing better data collection. After returning to the scanner, they completed the remaining 2 tasks and diffusion tensor imaging sequence. Before and after every acquisition, the caregiver RA communicated with the child through an intercom system embedded in the child’s noise-canceling headphones, to remind the child to stay still and assess their ability to continue through the study. The child was told that if they remained still enough during the entirety of the scan session, they would receive a big prize which they had selected before entering the scanner. After each scan, the caregiver RA communicated progress toward this goal.

The emotion-regulation task included images of children expressing emotional distress or in potentially dangerous situations (eg, being physically threatened by an adult). Following completion of this task, the child RA completed a debrief with the child to assure them that all people in the pictures were “playing-pretend” for the pictures. Next, the child RA completed 2 interviews with the child to assess their experiences of neglect and interpersonal violence in a separate space from the caregiver. If abuse or neglect were suspected based on the child’s endorsements, the RAs consulted with a staff clinical psychologist or the study principal investigator to evaluate if mandatory reporting thresholds were met and gather relevant information for appropriate child protection agencies. All participating caregivers were offered a consultation with a clinical psychologist following their child’s visit to understand how they had performed on standardized tests and interviews.

#### Neuroimaging Protocol With Young Children

At the end of visit 2, the child completed a mock MRI scan to prepare them for their final study visit. The child RA first introduced the child to the mock scan using a picture book that described the MRI machine, its function, and the importance of staying still. The child RA played scanner sounds for the child and showed the child the earplugs and headphones. This was a way to help the child acclimate to the sounds making them as normal as everyday items, such as a telephone ringing or a truck’s horn. The child then sat on the bed of the mock MRI machine and looked inside of the mock scan to see the screen they would watch their movie on. The child RA helped the child insert the earplugs into their ears, placed the headphones over their ears, and the child was asked to lie down and remain as still as possible while they watched a video for about 5 minutes. If the child expressed discomfort or asked to stop anytime during the process, the child RA would respond accordingly. For children who were fearful of the scan, extra steps were taken to assess whether the child was willing and able to try again. For children who were willing to try again, they completed the mock scan loading procedure again.

During visit 3, the child was asked to pick out prizes they would want to win after playing games. The child completed a practice round and then completed the test of the inhibitory control task. Once the game was completed, the child received their prize and were told that they would play a similar game. They were also informed that they would only be practicing this version of the game because the real game (test) would be played later in the scanner. The child then chose the prize they would like to win after completing the task in the MRI. Behavioral notes were recorded by the RA on the child’s hand use, performance, and prize selection. If the child exhibited a lack of understanding of the game rules, the RA would correct them and allow them to practice again. The next task, a reward sensitivity task, was introduced to the child. The child chose another small prize they would want to win for this game. The final task training was an emotion regulation task. Before completion of this training, caregivers were shown pictures of the training and test stimuli that the child would see so they could specifically consent to their child being shown this mildly negative content. If a caregiver preferred for their child to not see select images, those images were removed from training and test (caregivers could also opt out of this game). Once the child completed emotion regulation task training, they chose a medium-sized prize to win for once they completed the game in the scanner and began to practice the task.

After completion of all task training, the child was told about the special game buttons they would use in the scan to play their games. Then, the child was given a sheet with Disney movies and asked to pick a movie that they would like to watch during the MPRAGE scan. They also picked out a snack for their snack break and a small prize for watching their movie. Finally, the child was reminded about the importance of staying still in the scanner and introduced to “staying still points.” The child was informed that throughout the scan they could win points for staying still to win a large prize. If they won enough points by staying still throughout the scan, they could go home with the large prize they selected. In addition, the child was reminded that the child RA would be standing next to them during the duration of the scan while another team member (caregiver RA) would be talking to them through their headphones. The child RA also informed the child that to help them win their “staying still points,” they would put their hand on the child’s leg to remind them to stay still if they were moving. The children were given all the prizes they selected during this training phase, regardless of task performance or actual movement in the scanner.

Once all prizes were selected and instructions were given to the child, the child met their caregiver at the MRI suite. The research staff provided the caregiver with scrubs to help their child change, and their pediatric MRI screening form was overviewed again with the caregiver by the MRI technician. The child’s height and weight were measured by staff and reported to the technician. The child then entered the scan room with the child RA. The child RA and MRI tech walked the child through all the materials mimicking their mock scan for a sense of familiarity. In addition, the child RA showed the child their scan buttons and instructed them on how to use them. The headphone volume was tested before putting them over the child’s ears, and the child was introduced to the voice of caregiver RA at the scan console and asked to respond verbally to them.

### Measures

#### Early Adversity

Caregivers and children reported on adverse experiences involving deprivation, threat, and unpredictability using self-report measures and interviews. Continuous composite scores representing the severity of deprivation, threat, and unpredictability will be calculated by summing sample-standardized scores from the self-report and observational measures and by creating count scores reflecting the number of different exposures to deprivation, threat, and unpredictability. Deprivation will be operationalized as exposure to neglect and low social and cognitive stimulation. Exposure to neglect is reported by caregivers on the neglect subscale of the Conflict Tactics Scale (CTS) parent-child version [23], Juvenile Victimization Questionnaire [24], and exposure to physical, supervisory, and cognitive neglect reported by children on the Multidimensional Neglectful Behavior Scale [25]. Exposure to low cognitive stimulation was observed in the Home Observation Measurement of the Environment [26], reported by caregivers on the StimQ [27], and reported by children on the Multidimensional Neglectful Behavior Scale. In addition, indexes of caregiver use of complex language will be measured during the book reading PCI and included in some measures of deprivation pending factor analytic and other clustering approaches to dimension development.

Threat will be operationalized as exposure to physical abuse, sexual abuse, domestic violence, community violence, and other interpersonal violence. These exposures were reported by caregivers on the CTS parent-child version, CTS 2nd edition [28], Juvenile Victimization Questionnaire, University of California at Los Angeles Posttraumatic Stress Disorder Reaction Index [29], the Life Events Scale for Children [30], and the School Safety Questionnaire [31], and by children on the Violence Exposure Scale for Children-Revised [32]. Corporal punishment and endorsements of harsh punishment were additionally observed or reported by caregivers on the Home Observation Measurement of the Environment instrument.

Unpredictability will be operationalized as exposure to lack of consistent responding, instability of the family and physical environment, and disorganization reported by caregivers on the Family Life Project Chaos Scale [33], the Confusion, Hubub, and Order Scale [34], the US Department of Agriculture Food Insecurity Scale [35], the Family Routines Inventory [36], and the Questionnaire of Unpredictability in Childhood [37]. In addition, observations of entropy in caregiver behavior from the PCI tasks will be incorporated [38] into indexes of unpredictability pending factor analysis and other clustering approaches to dimension development.

#### Childhood Psychopathology

Child psychopathology was assessed using the Mini-International Neuropsychiatric Interview for Children and Adolescents and caregiver report on the Child Behavior Checklist [39-42].

Study questionnaires and interviews were administered to caregivers using Qualtrics [43] across the multiple study visits and took around 10 hours to complete in total. Moreover, all of these questionnaires and surveys have been validated and used in multiple studies exploring the impact of childhood adversity on child development, including various studies from our group [44-47]. For information about each individual measure’s validity and reliability please review its citation.

#### Fear Learning Task

The task involves 3 phases: habituation, learning, and extinction, and it is based on previous work studying fear learning in young children and work in adults examining fear learning to images of close friends [44,48-51]. On a computer screen, 4 different images, 2 of caregivers and 2 of strangers, were shown one at a time, 4 times each to the child (preacquisition). Then, the images were shown again and 2 of the pictures (caregiver and stranger) were paired with an aversive 95 dB noise, which was counterbalanced across participants (conditioning). The conditioning phase involves 6 to 10 trials of each face. The alarm noise rings for 2 stimuli (caregiver and stranger) but not for the other 2 stimuli (secondary caregiver and stranger). Finally, the pictures (caregiver or stranger) were shown one at a time again with no noises for 6 to 10 trials each (extinction). Children rated how much they fear or like each picture at the end of each phase. Children were informed that they can discontinue the task at any time. Skin conductance and heart rate were collected continuously throughout the task using MindWare Technologies. To measure skin conductance, 2 electrodes were attached to the index and middle finger of the child’s nondominant hand after the fingers were cleaned with rubbing alcohol. To measure continuous echocardiogram, we used MindWare Technologies BioLab acquisition software at 500 Hz. Two electrodes were attached to the participant’s collar bone and rib cage. Electrode attachment was always done in the presence of 2 RAs using electrodes designed for use with small children.

#### Parent-Child Observed Emotion Regulation

The researcher collected blood spot samples from the child, and then later from the caregiver, by pricking a finger with a pressure-activated lancet. For this procedure, the researcher set up the materials (eg, lancet, blood spot collection paper, alcohol wipe, gauze, and Band-Aids) and then verbally described the finger prick to the caregiver and child. Each child was first instructed to shake their hand toward the floor. The researcher then gently massaged the finger, cleaned the puncture site with an alcohol wipe, pricked the finger with the pressure-activated lancet, and held the child’s finger to place 2 to 5 drops of blood on the collection paper. After obtaining the samples, the researcher cleaned up the materials and left the space, while the caregiver was asked to place a Band-Aid around the child’s finger. Throughout the finger prick procedure, caregivers were welcome to speak and interact with their child and reposition themselves or their child (eg, taking child in lap) however they wished. No instructions were provided to caregivers on how to respond to their child.

The finger prick also served as the context for the coding of caregiver- and child-directed behaviors using a novel coding scheme based on existing schemes [22,52,53]. The full finger prick blood collection procedure was video recorded, with cameras placed in a location that captured both caregiver and child. Children’s emotion regulation, caregivers’ sensitivity, caregivers’ hostility, and the dyad’s level of engagement were coded from the video-recordings on scales of 1 to 5. Child distress was rated on a scale of 1 to 5 every 30 seconds of the video. Furthermore, the presence or absence of children’s engagement in specific behaviors (eg, positive expressions, distraction, and avoidance), caregivers’ initiation of strategies to regulate the child (eg, distraction, calming techniques, and emotion word use), and dyadic behavioral mimicry were also coded every 30 seconds. The full finger-prick procedure from the start to end times of coding ranged from 2 minutes and 30 seconds to >5 minutes, depending on both the speed of the researcher and child’s level of discomfort. Videos where caregivers and children had instances of speaking in languages other than English (eg, Spanish, Mandarin, and Russian) were translated by multilingual RAs on the study team before coding. Each video was coded by at least 2 individuals on the team to maintain reliability and increase coding accuracy.

#### Structural MRI and fMRI

Imaging acquisition was performed on a Siemens 3T Prisma scanner with a 32-channel head coil. T1-weighted MPRAGE were acquired for anatomical coregistration with fMRI and for analysis of cortical thickness, surface area, subcortical volume, and T1/T2*-weighted mapping (repetition time=2500 ms, echo time=2.88 ms, flip angle=8°, field of view=256 mm^2^, 176 slices, in-plane voxel size=1 mm^3^). For task blood-oxygenation–level dependent signal is acquired using a gradient-echo T2*-weighted echo-planar imaging (EPI) sequence. Seventy-two 2 mm slices are acquired positioned parallel to the anterior commissure-posterior commissure line. Imaging parameters are repetition time=0.8 seconds, echo time=37 ms, flip angle=52°, bandwidth=2300, echo spacing=0.58 ms, filed of view=104×104, and interleaved slice acquisition, multiband acceleration factor is set to 8. We used FIRMM software to monitor movement in a moment-to-moment fashion, allowing us to give real time feedback to participants and adjust scanning appropriately. We use AAScout to position slices on the long axis of the brain. AAScout was run before acquiring data at the beginning of the scan, following the break for a snack, and anytime a substantial movement occurred. All slice acquisition for EPI scans came from the AAScout. Using this autoalign procedure limited our ability to place slices on the AC-PC line but it allowed us to acquire EPI scans across runs and after the child exited and entered the scanner using identical slice placement on the child’s brain.

T1-weighted scans will be used to calculate cortical thickness and gray matter volumes using FreeSurfer. Automatic image segmentation is used to identify subcortical gray matter structures. Next, gray and white matter, and gray matter and cerebrospinal fluid boundaries are constructed, and the cortex is parcellated based on the structure of gyri and sulci. The results will be manually inspected and edited. These procedures have demonstrated good test-retest reliability across scanner manufacturers and field strengths, and cortical thickness measures have been validated against manual measurement [54]. Preprocessing and statistical analysis will be performed using fMRI data preprocessing pipeline [55] and Functional Magnetic Resonance Imaging of the Brain Software Library (FSL) [56] to capitalize on the relative strengths of different statistical packages. Preprocessing will include spatial realignment, slice-time correction (4D reconstruction), and spatial smoothing. Outliers (frame wise displacement <0.9 mm for task, <0.2 mm for rest), motion regressors and their derivatives (6 rigid body parameters), cerebrospinal fluid, and white matter signal will be included in person-level models. After estimating the person-level models, contrast images will be normalized into standard anatomical space using an appropriate atlas. FSL will be used to construct generalized linear models at the level of the run, which will be combined using FLAME to the level of the person and group.

#### Executive Function fMRI Task

We assessed executive function using the conditioned appetitive response inhibition task. A total of 1 version of this task is commonly used in early childhood [57] and another in middle childhood, adolescence, and adulthood [58-61]. Performance and neural recruitment in this task has been linked with transdiagnostic risk for psychopathology [61]. In the training period of the conditioned appetitive response inhibition task (outside the scanner), participants are rewarded for pressing a button when they see a specific stimulus. In the scanner participants perform a go-no go task where 30% of the stimuli are “no go” and 70% are “go” stimuli. Within the “no go” stimuli, half are the stimulus which was rewarded during training and half are a previously seen but not rewarded stimulus. This task was administered in 2 runs lasting about 5 minutes each.

#### Reward fMRI Task

For the reward task, participants see a question mark and are asked to guess what is “behind” the question mark by pressing a button. After responding, participants receive feedback: either gold coins for guessing correctly or gold coins with an “X” through them for guessing incorrectly. The feedback is predetermined and randomly assigned such that half of responses will be “correct,” and thus, the child will receive a reward, and the other half will be “incorrect,” and the child will garner a loss. Children were shown before each trial if it was a high value (win US $1.00, lose US $0.50) or a low value (win US $0.20, lose US $0.10) trial [62]. Although wins and losses were reported as monetary amounts on each trial, the child was told that they would not receive money but instead earned a prize.

#### Emotion Regulation fMRI Task

We assessed emotion reactivity and regulation using a novel task developed for this study, which is a variant of a commonly used older-child and adult cognitive-reappraisal task [63,64]. This novel task is optimized for use in early childhood. In older child and adult cognitive-reappraisal tasks, participants are told to use reappraisal strategies, or stories, to distance themselves from negative stimuli. In the novel early childhood version, participants are *told* reappraisal stories by an adult instead of coming up with these stories on their own, which mimics strategies regularly used by caregivers to help children regulate their emotions. Versions of this task have been used previously in EEG studies [63,65].

### Analytic Plan

Associations between EA and task performance will be assessed using linear regression. All analyses will include controls for age, sex, cognitive ability, and aspects of family environment not directly related to deprivation and threat, such as caregiver education. Differences in cortical thickness as a function of deprivation, threat, and unpredictability will be examined using surface-based analysis tools in Free Surfer, correcting for multiple comparisons using false discovery rate. To examine the influence of deprivation, we will estimate regressions to test for linear associations between deprivation exposure and cortical thickness.

fMRI data analysis will be conducted in FSL. Stimulus onset regressors will be convolved with a canonical hemodynamic response function in all tasks. The general linear model will be estimated in individual space, and nuisance regressors will be included for motion parameters and outliers. Individual-level regression coefficients will be submitted to group-level random effects models, and linear regression parameters will be estimated to examine activation as a function of deprivation and threat. Cluster-wise false positive rates of *P*<.05 corrected for multiple comparisons will be applied. Finally, to determine whether the impact of deprivation threat, or unpredictability on neural structure and function are pathways linking EA to psychopathology, we will test a multiple mediation model.

### Ethical Considerations

This study has institutional review board approval from The University of North Carolina at Chapel Hill (study 16-1278). Informed consent was obtained from all individual participants included in the study. Participants received US $280 in total across the 3 study visits for their time and effort. Participating children earned prizes by completing study tasks. In addition, families were reimbursed for transportation costs associated with attending study visits.

## Results

### Overview

The WHALE study is funded by the National Institute of Mental Health (R01MH115004; see grant review provided in Multimedia Appendix 2). The study began data collection in November 2018 and continued through May 2024. Recruitment for this study has concluded, and data collection is ongoing. Study results will be available in 2024.

### Sample

Participants (N=301; 49% male) were enrolled in the study when they were aged between 4 years 0 months and 7 years 3 months. Average age at first visit was 5.69 (SD 0.83) years. Our sample is racially and ethnically diverse with 36.8% (111/301) self-identified as Black or African American, 15.2% (46/301) as other or mixed race, 6.9% (21/301) Asian, 0.9% (3/301) American Indian or Pacific Islander, 11.9% (36/301) Hispanic or Latino, and 36.8% (111/301) non-Hispanic White. Of the total participants who enrolled in the study, 26 (8.6%) did not participate in any study visits. Data collection occurred between November 2018 and May 2024; study attrition was primarily due to disruption in the operation of the laboratory during the COVID-19 pandemic.

### Early Adversity

The sample has substantial strengths. First, we have multi-informant assessment of exposure to adversity, and multimodal assessments of executive function and emotion dysregulation, including caregiver report, child task performance, and neural function. Second, we recruited participants based primarily on indexes of structural inequality. Consistent with evidence supporting a strong association between structural inequality and adverse childhood experiences, participating families reported a range of deprivation and threat experiences (Multimedia Appendix 3).

In addition, this sampling strategy more accurately reflects the range of profiles that emerge from exposure to adversity, which can be obscured when sampling is predicated on the outcome (eg, psychopathology) [66]. Finally, this sample was recruited during the peak age for likely exposure to adversity in the home. Evidence from epidemiological literature indicates that the early childhood period carries the greatest risk for exposure to maltreatment [67,68] and corporal punishment [69], and it is a period of maximal brain, cognitive, and emotional development [70]. Assessing neural function in this age range is rare. Here we have excellent measurement of cognitive, emotional, and neural functioning during this early period, and thus, the opportunity to observe the impact of adversity on child development.

## Discussion

### Principal Findings

The WHALE study is one of the first studies to explore the differential impact of deprivation, threat, and unpredictability on neural function and risk for psychopathology. Recruitment is complete with a sample of 301 participants, and our sampling strategy was successful in recruiting an ethnic and racially diverse sample. Moreover, in a subsample of participants (n=270), we observe a wide range of deprivation and threat experiences. These distributions are similar to prior research aiming to differentiate the effects of dimensions of adversity on neurodevelopment in prior samples [42,69], and will allow us to capture the unique effects of experience on development. As this is an ongoing study, no formal testing of the project’s hypotheses has been conducted yet.

Previous research documents the differential effects of deprivation and threat on psychopathology through distinct neurobiological mechanisms in adolescence [71-73]. However, no study has evaluated these associations in early childhood, despite consistent evidence that early exposure to adversity is associated with differences in neurodevelopment during this period [44,47]. To the best of our knowledge, the WHALE study is one of the first cohort studies exploring the differential effects of deprivation, threat, and unpredictability on neurobiological mechanisms that increase risk for psychopathology. The 3 primary aims of the study were to examine (1) the associations between deprivation, threat, cognitive control, and fear learning, (2) the association between deprivation and threat with psychopathology through its hypothesized neural mechanisms, and (3) the associations between unpredictability, reward learning, and psychopathology. The results of this study will provide unique insights into the neurobiological mechanisms by which EA increase risk for psychopathology.

### Limitations and Future Directions

One of the key limitations in this study is its cross-sectional design, thus future research should aim to explore the role of EA on neurodevelopmental outcomes longitudinally. These designs are important as they allow to establish directionality in observed effects. Another limitation includes the exclusion of other dimensions of EA that could have similar or differential effects on child development, such as parental loss [74]. Thus, future studies should aim to have a holistic assessment of children’s environments that can capture other dimensions of adversity. Our results will elucidate the effects separate dimensions of EA on child neurodevelopment.

### Conclusions

This protocol describes a study aiming to explore the roles of deprivation, threat, and unpredictability on neurodevelopment. We were successful at enrolling 301 children and their families into the study, and obtaining measurement on children’s exposure to EA, neural function, and psychopathology. The results from this study could inform future intervention and prevention strategies to aid children exposed to EA.

## Appendix

Multimedia Appendix 1 Overview of study measures.

Multimedia Appendix 2 Peer-review report from the Child Psychopathology and Developmental Disabilities Study Section (CPDD) - Biobehavioral and Behavioral Processes Integrated Review Group - Center for Scientific Review (National Institutes of Health, USA).

Multimedia Appendix 3 Histograms of deprivation and threat composite scores in a subsample of participants (n=270). The number of subdomains endorsed for physical neglect, insufficient supervision, insufficient resources, cognitive deprivation, and physical environment (0-5) are shown for deprivation. The number of subdomains endorsed for physical abuse, peer and community violence, sexual abuse, domestic violence, emotional abuse, and other threatening experiences (0-6) are shown for threat.

## Abbreviations

CTS: Conflict Tactics Scale
DMAP: dimensional model of adversity and psychopathology
EA: early adversity
EEG: electroencephalogram
fMRI: functional magnetic resonance imaging
FSL: Functional Magnetic Resonance Imaging of the Brain Software Library
MPRAGE: magnetization-prepared rapid gradient echo
MRI: magnetic resonance imaging
PCI: parent-child interaction
RA: research assistant
WHALE: Wellness Health and Life Experiences

## Data Availability

The data sets generated from this study are not publicly available because of ongoing data collection efforts.

## References

[1] McLaughlin KA, Greif Green J, Gruber MJ, Sampson NA, Zaslavsky AM, Kessler RC (2012). Childhood adversities and first onset of psychiatric disorders in a national sample of US adolescents. Arch Gen Psychiatry.

[2] Green JG, McLaughlin KA, Berglund PA, Gruber MJ, Sampson NA, Zaslavsky AM, Kessler RC (2010). Childhood adversities and adult psychiatric disorders in the national comorbidity survey replication I: associations with first onset of DSM-IV disorders. Arch Gen Psychiatry.

[3] Kessler RC, McLaughlin KA, Green JG, Gruber MJ, Sampson NA, Zaslavsky AM, Aguilar-Gaxiola S, Alhamzawi AO, Alonso J, Angermeyer M, Benjet C, Bromet E, Chatterji S, de Girolamo G, Demyttenaere K, Fayyad J, Florescu S, Gal G, Gureje O, Haro JM, Hu C, Karam EG, Kawakami N, Lee S, Lépine JP, Ormel J, Posada-Villa J, Sagar R, Tsang A, Ustün TB, Vassilev S, Viana MC, Williams DR (2010). Childhood adversities and adult psychopathology in the WHO World Mental Health surveys. Br J Psychiatry.

[4] Woodward LJ, Fergusson DM (2001). Life course outcomes of young people with anxiety disorders in adolescence. J Am Acad Child Adolesc Psychiatry.

[5] Greenberg PE, Sisitsky T, Kessler RC, Finkelstein SN, Berndt ER, Davidson JRT, Ballenger JC, Fyer AJ (1999). The economic burden of anxiety disorders in the 1990s. J Clin Psychiatry.

[6] Sheridan MA, McLaughlin KA (2014). Dimensions of early experience and neural development: deprivation and threat. Trends Cogn Sci.

[7] Sheridan MA, Fox NA, Zeanah CH, McLaughlin KA, Nelson CA (2012). Variation in neural development as a result of exposure to institutionalization early in childhood. Proc Natl Acad Sci U S A.

[8] Opendak M, Gould E, Sullivan R (2017). Early life adversity during the infant sensitive period for attachment: programming of behavioral neurobiology of threat processing and social behavior. Dev Cogn Neurosci.

[9] Anda RF, Felitti VJ, Bremner JD, Walker JD, Whitfield C, Perry BD, Dube SR, Giles WH (2006). The enduring effects of abuse and related adverse experiences in childhood. A convergence of evidence from neurobiology and epidemiology. Eur Arch Psychiatry Clin Neurosci.

[10] Chapman DP, Whitfield CL, Felitti VJ, Dube SR, Edwards VJ, Anda RF (2004). Adverse childhood experiences and the risk of depressive disorders in adulthood. J Affect Disord.

[11] Felitti VJ (2009). Adverse childhood experiences and adult health. Acad Pediatr.

[12] Gunnar M, Quevedo K (2007). The neurobiology of stress and development. Annu Rev Psychol.

[13] Bradley RH, Mundfrom DJ, Whiteside L, Caldwell BM, Casey PH, Kirby RS, Hansen S (1994). A reexamination of the association between HOME scores and income. Nursing Research.

[14] Vogel SC, Perry RE, Brandes-Aitken A, Braren S, Blair C (2021). Deprivation and threat as developmental mediators in the relation between early life socioeconomic status and executive functioning outcomes in early childhood. Dev Cogn Neurosci.

[15] Mumford JA (2012). A power calculation guide for fMRI studies. Soc Cogn Affect Neurosci.

[16] Sobie EA (2011). An introduction to MATLAB. Sci Signal.

[17] Muthén LK, Muthén BO (2019). Mplus user's guide (1998-2019). Muthén & Muthén.

[18] Muthén B, Asparouhov T (2003). Modeling interactions between latent and observed continuous variables using maximum-likelihood estimation in Mplus. Muthén & Muthén.

[19] Muthén LK, Muthén BO (2002). How to use a Monte Carlo study to decide on sample size and determine power. Struct Equ Modeling Multidiscip J.

[20] Slopen N, Koenen KC, Kubzansky LD (2014). Cumulative adversity in childhood and emergent risk factors for long-term health. J Pediatr.

[21] Weissman DG, Rosen ML, Colich NL, Sambrook KA, Lengua LJ, Sheridan MA, McLaughlin KA (2022). Exposure to violence as an environmental pathway linking low socioeconomic status with altered neural processing of threat and adolescent psychopathology. J Cogn Neurosci.

[22] NICHD Early Child Care Research Network (1999). Child care and mother-child interaction in the first 3 years of life. Dev Psychol.

[23] Straus MA, Hamby SL, Finkelhor D, Moore DW, Runyan D (1998). Identification of child maltreatment with the parent-child conflict tactics scales: development and psychometric data for a national sample of American parents. Child Abuse Negl.

[24] Finkelhor D, Ormrod R, Turner H, Hamby SL (2005). The victimization of children and youth: a comprehensive, national survey. Child Maltreat.

[25] Kantor GK, Holt MK, Mebert CJ, Straus MA, Drach KM, Ricci LR, MacAllum CA, Brown W (2004). Development and preliminary psychometric properties of the multidimensional neglectful behavior scale-child report. Child Maltreat.

[26] Mott FL (2004). The utility of the HOME-SF scale for child development research in a large national longitudinal survey: the national longitudinal survey of youth 1979 cohort. Parenting.

[27] Dreyer BP, Mendelsohn AL, Tamis‐LeMonda CS (1996). Assessing the child's cognitive home environment through parental report; reliability and validity. Early Dev Parent.

[28] Straus MA, Hamby SL, Boney-McCoy S, Sugarman DB (2016). The revised conflict tactics scales (CTS2): development and preliminary psychometric data. J Fam Issues.

[29] Steinberg AM, Brymer MJ, Kim S, Briggs EC, Ippen CG, Ostrowski SA, Gully KJ, Pynoos RS (2013). Psychometric properties of the UCLA PTSD reaction index: part I. J Trauma Stress.

[30] LONGSCAN child’s life events [instrument adapted from the coddington child life events scales]. LONGSCAN.

[31] Hunter WM, Cox CE, Teagle S, Johnson RM, Mathew R, Knight ED, Leeb RT, Smith JB (2003). Measures for assessment of functioning and outcomes in longitudinal research on child abuse. LONGSCAN Coordinating Center, University of North Carolina at Chapel Hill.

[32] Raviv A, Erel O, Fox NA, Leavitt LA, Raviv A, Dar I, Shahinfar A, Greenbaum CW (2001). Individual measurement of exposure to everyday violence among elementary schoolchildren across various settings. J Community Psychol.

[33] Vernon-Feagans L, Garrett-Peters P, Willoughby M, Mills-Koonce R, The Family Life Project Key Investigators (2012). Chaos, poverty, and parenting: predictors of early language development. Early Child Res Q.

[34] Matheny AP, Wachs TD, Ludwig JL, Phillips K (1995). Bringing order out of chaos: psychometric characteristics of the confusion, hubbub, and order scale. J Appl Dev Psychol.

[35] Blumberg SJ, Bialostosky K, Hamilton WL, Briefel RR (1999). The effectiveness of a short form of the Household Food Security scale. Am J Public Health.

[36] Jensen EW, James SA, Boyce WT, Hartnett SA (1983). The family routines inventory: development and validation. Soc Sci Med.

[37] Glynn L, Stern H, Howland M, Risbrough V, Baker D, Nievergelt C, Baram T, Davis E (2019). Measuring novel antecedents of mental illness: the Questionnaire of Unpredictability in childhood. Neuropsychopharmacology.

[38] Davis EP, Stout SA, Molet J, Vegetabile B, Glynn LM, Sandman CA, Heins K, Stern H, Baram TZ (2017). Exposure to unpredictable maternal sensory signals influences cognitive development across species. Proc Natl Acad Sci U S A.

[39] Achenbach TM, Rescorla L (2001). Manual for the ASEBA School-Age Forms & Profiles: Child Behavior Checklist for Ages 6-18, Teacher’s Report Form, Youth Self-Report.

[40] Achenbach TM, Ruffle TM (2000). The child behavior checklist and related forms for assessing behavioral/emotional problems and competencies. Pediatr Rev.

[41] Nock MK, Holmberg EB, Photos VI, Michel BD (2007). Self-injurious thoughts and behaviors interview: development, reliability, and validity in an adolescent sample. Psychol Assess.

[42] Sheehan DV, Sheehan KH, Shytle RD, Janavs J, Bannon Y, Rogers JE, Milo KM, Stock SL, Wilkinson B (2010). Reliability and validity of the mini international neuropsychiatric interview for children and adolescents (MINI-KID). J Clin Psychiatry.

[43] Go all-in on making every experience count. Qualtrics.

[44] Machlin L, Miller AB, Snyder J, McLaughlin KA, Sheridan MA (2019). Differential associations of deprivation and threat with cognitive control and fear conditioning in early childhood. Front Behav Neurosci.

[45] Miller AB, Sheridan MA, Hanson JL, McLaughlin KA, Bates JE, Lansford JE, Pettit GS, Dodge KA (2018). Dimensions of deprivation and threat, psychopathology, and potential mediators: a multi-year longitudinal analysis. J Abnorm Psychol.

[46] Lambert HK, King KM, Monahan KC, McLaughlin KA (2016). Differential associations of threat and deprivation with emotion regulation and cognitive control in adolescence. Dev Psychopathol.

[47] Machlin L, Egger HL, Stein CR, Navarro E, Carpenter KL, Goel S, Patel KK, Copeland WE, Sheridan MA (2023). Distinct associations of deprivation and threat with alterations in brain structure in early childhood. J Am Acad Child Adolesc Psychiatry.

[48] Jovanovic T, Nylocks KM, Gamwell KL, Smith A, Davis TA, Norrholm SD, Bradley B (2014). Development of fear acquisition and extinction in children: effects of age and anxiety. Neurobiol Learn Mem.

[49] Eisenberger NI, Master SL, Inagaki TK, Taylor SE, Shirinyan D, Lieberman MD, Naliboff BD (2011). Attachment figures activate a safety signal-related neural region and reduce pain experience. Proc Natl Acad Sci U S A.

[50] Hornstein EA, Fanselow MS, Eisenberger NI (2016). A safe haven: investigating social-support figures as prepared safety stimuli. Psychol Sci.

[51] Hornstein EA, Eisenberger NI (2017). Unpacking the buffering effect of social support figures: social support attenuates fear acquisition. PLoS One.

[52] Hagstrøm J, Spang KS, Christiansen BM, Maigaard K, Vangkilde S, Esbjørn BH, Jepsen JR, Plessen KJ (2019). The puzzle of emotion regulation: development and evaluation of the tangram emotion coding manual for children. Front Psychiatry.

[53] Wakschlag LS, Hill C, Carter AS, Danis B, Egger HL, Keenan K, Leventhal BL, Cicchetti D, Maskowitz K, Burns J, Briggs-Gowan MJ (2008). Observational assessment of preschool disruptive behavior, part I: reliability of the disruptive behavior diagnostic observation schedule (DB-DOS). J Am Acad Child Adolesc Psychiatry.

[54] Fischl B, Salat DH, Busa E, Albert M, Dieterich M, Haselgrove C, van DK, Killiany R, Kennedy D, Klaveness S, Montillo A, Makris N, Rosen B, Dale AM (2002). Whole brain segmentation: automated labeling of neuroanatomical structures in the human brain. Neuron.

[55] Esteban O, Markiewicz CJ, Blair RW, Moodie CA, Isik AI, Erramuzpe A, Kent JD, Goncalves M, DuPre E, Snyder M, Oya H, Ghosh SS, Wright J, Durnez J, Poldrack RA, Gorgolewski KJ (2019). fMRIPrep: a robust preprocessing pipeline for functional MRI. Nat Methods.

[56] Fagiolo G, Waldman A, Hajnal JV (2008). A simple procedure to improve FMRIb software library brain extraction tool performance. Br J Radiol.

[57] Winter W, Sheridan M (2014). Previous reward decreases errors of commission on later 'No-Go' trials in children 4 to 12 years of age: evidence for a context monitoring account. Dev Sci.

[58] Davidow JY, Sheridan MA, Van Dijk KR, Santillana RM, Snyder J, Vidal Bustamante CM, Rosen BR, Somerville LH (2019). Development of prefrontal cortical connectivity and the enduring effect of learned value on cognitive control. J Cogn Neurosci.

[59] Meyer KN, Sheridan MA, Hopfinger JB (2020). Reward history impacts attentional orienting and inhibitory control on untrained tasks. Atten Percept Psychophys.

[60] Rodriguez-Thompson AM, Meyer KM, Davidow JY, Van Dijk KR, Santillana RM, Snyder J, Vidal Bustamante CM, Hollinshead MO, Rosen BR, Somerville LH, Sheridan MA (2020). Examining cognitive control and reward interactions in adolescent externalizing symptoms. Dev Cogn Neurosci.

[61] Rodriguez-Thompson AM, Miller AB, Wade M, Meyer KN, Machlin L, Bonar AS, Patel KK, Giletta M, Hastings PD, Nock MK, Rudolph KD, Slavich GM, Prinstein MJ, Sheridan MA (2024). Neural correlates of the p factor in adolescence: cognitive control with and without enhanced positive affective demands. Biol Psychiatry Cogn Neurosci Neuroimaging.

[62] Somerville LH, Bookheimer SY, Buckner RL, Burgess GC, Curtiss SW, Dapretto M, Elam JS, Gaffrey MS, Harms MP, Hodge C, Kandala S, Kastman EK, Nichols TE, Schlaggar BL, Smith SM, Thomas KM, Yacoub E, Van Essen DC, Barch DM (2018). The lifespan human connectome project in development: a large-scale study of brain connectivity development in 5-21 year olds. Neuroimage.

[63] Dougherty LR, Blankenship SL, Spechler PA, Padmala S, Pessoa L (2015). An fMRI pilot study of cognitive reappraisal in children: divergent effects on brain and behavior. J Psychopathol Behav Assess.

[64] Ochsner KN, Silvers JA, Buhle JT (2012). Functional imaging studies of emotion regulation: a synthetic review and evolving model of the cognitive control of emotion. Ann N Y Acad Sci.

[65] Myruski S, Birk S, Karasawa M, Kamikubo A, Kazama M, Hirabayashi H, Dennis-Tiwary T (2019). Neural signatures of child cognitive emotion regulation are bolstered by parental social regulation in two cultures. Soc Cogn Affect Neurosci.

[66] Tyrer S, Heyman B (2016). Sampling in epidemiological research: issues, hazards and pitfalls. BJPsych Bull.

[67] Finkelhor D, Ormrod RK, Turner HA (2009). The developmental epidemiology of childhood victimization. J Interpers Violence.

[68] Finkelhor D, Saito K, Jones L (2014). Updated trends in child maltreatment. Crimes Against Children Research Center.

[69] Finkelhor D, Turner H, Wormuth BK, Vanderminden J, Hamby S (2019). Corporal punishment: current rates from a national survey. J Child Fam Stud.

[70] Tsujimoto S (2008). The prefrontal cortex: functional neural development during early childhood. Neuroscientist.

[71] Weissman DG, Jenness JL, Colich NL, Miller AB, Sambrook KA, Sheridan MA, McLaughlin KA (2020). Altered neural processing of threat-related information in children and adolescents exposed to violence: a transdiagnostic mechanism contributing to the emergence of psychopathology. J Am Acad Child Adolesc Psychiatry.

[72] McLaughlin KA, Hatzenbuehler ML, Mennin DS, Nolen-Hoeksema S (2011). Emotion dysregulation and adolescent psychopathology: a prospective study. Behav Res Ther.

[73] McLaughlin KA, Sheridan MA, Gold AL, Duys A, Lambert HK, Peverill M, Heleniak C, Shechner T, Wojcieszak Z, Pine DS (2016). Maltreatment exposure, brain structure, and fear conditioning in children and adolescents. Neuropsychopharmacology.

[74] McGinnis EW, Sheridan M, Copeland WE (2022). Impact of dimensions of early adversity on adult health and functioning: a 2-decade, longitudinal study. Dev Psychopathol.

