# Supplementary material for: Testing a Conceptual Model of Early Adversity, Neural Function, and Psychopathology: Protocol for a Retrospective Observational Cohort Study
Source: JMIR Res Protoc. 2024 Sep 17;13:e59636. doi: 10.2196/59636 (PMC11445632; doi:10.2196/59636)
Supplement: Multimedia Appendix 1 [file resprot_v13i1e59636_app1.docx]

**Multimedia Appendix 1.**

*Measures Administered across Data Collection Visits*

| Measure | Type | Respondent | Visit 1 | Visit 2 | Visit 3 |
| --- | --- | --- | --- | --- | --- |
| Dimensional Change Card Short | T | C | ✓ |  |  |
| Executive Function (EF) Table Tasks | T | C | ✓ |  |  |
| Class Task | T | C | ✓ |  |  |
| Ladder Task | T | C | ✓ |  |  |
| Pinata Task | T | C | ✓ |  |  |
| SIMON Task | T | C | ✓ |  |  |
| Home Observation Measurement of the Environment (HOME) | I | PC | ✓ |  |  |
| Developmental and Behavioral Pediatrics Intake Questionnaire | I | PC | ✓ |  |  |
| LONGSCAN Demographic Questionnaires (C & PC versions) | S | PC | ✓ |  |  |
| Family Composition Questionnaire (Project Developed) | S | PC | ✓ |  |  |
| Parental Incarceration Questionnaire | S | PC | ✓ |  |  |
| Pittsburgh Sleep Quality Index | S | PC | ✓ |  |  |
| Temporary Housing Questionnaire (Project Developed) | S | PC | ✓ |  |  |
| Adoption/Foster Care Status (Project Developed) | S | PC | ✓ |  |  |
| Language Inquiry Questionnaire (Project Developed) | S | PC | ✓ |  |  |
| School Safety Questionnaire | S | PC | ✓ |  |  |
| Child Life Events Scale for Children | S | PC | ✓ |  |  |
| Daycare Questionnaire (Project Developed) | S | PC | ✓ |  |  |
| Family Life Project CHAOS Questionnaire | S | PC | ✓ |  |  |
| Use of burner phones (Project Developed) | S | PC | ✓ |  |  |
| Kaufman Brief Intelligence Test Second Edition | T | C |  | ✓ |  |
| Fear Conditioning Task | T | C |  | ✓ |  |
| Parent-Child Interaction Tasks | T | C & PC |  | ✓ |  |
| Resting EEG | T | C |  | ✓ |  |
| EEG Frustration Task (Project Developed) | T | C |  | ✓ |  |
| Puppet Task | T | C |  | ✓ |  |
| Emotion Word Definition Task | T | C |  | ✓ |  |
| Caregiver Pinata Task | T | C |  | ✓ |  |
| Saliva | T | C |  | ✓ |  |
| Dried Blood Spot | T | C |  | ✓ |  |
| Mini-International Psychiatric Interview | I | PC |  | ✓ |  |
| Stim-Q Cognitive Home Environment Questionnaire | I | PC |  | ✓ |  |
| Child Self-Injurious and Behaviors Interview | I | PC |  | ✓ |  |
| Adult Self-Injurious and Behaviors Interview | I | PC |  | ✓ |  |
| Family Relationship Interview (Project Developed) | I | PC |  | ✓ |  |
| Conflict and Tactics Scale – 2 | S | PC |  | ✓ |  |
| Child Abuse Potential Inventory | S | PC |  | ✓ |  |
| Personal Relative Deprivation Measure | S | PC |  | ✓ |  |
| Food Insecurity | S | PC |  | ✓ |  |
| Childhood Trauma Questionnaire | S | PC |  | ✓ |  |
| Confusion, Hubub, and Order Scale | S | PC |  | ✓ |  |
| Family Adaptability and Cohesion Evaluation Scale IV | S | PC |  | ✓ |  |
| Adult Self Report from ASEBA | S | PC |  | ✓ |  |
| Parental Social Class Socialization Measure | S | PC |  | ✓ |  |
| MacArthur Survey | S | PC |  | ✓ |  |
| Conflict Tactic Scales Parent-Child Version | S | PC |  | ✓ |  |
| Juvenile Victimization Questionnaire Second Revision | S | PC |  | ✓ |  |
| UCLA Post-traumatic Stress Disorder Index | S | PC |  | ✓ |  |
| Child and Adult Mental Health Service Utilization | S | PC |  | ✓ |  |
| Child Behavior Checklist | S | PC |  | ✓ |  |
| Centers for Disease Control Lead Exposure Survey | S | PC |  | ✓ |  |
| Emotion Regulation fMRI Task | T | C |  |  | ✓ |
| Conditioned Approach Response Inhibition fMRI Task | T | C |  |  | ✓ |
| Reward fMRI Task | T | C |  |  | ✓ |
| Structural scan, MPRAGE T1-weighted imaging | T | C |  |  | ✓ |
| Diffusion tensor imaging | T | C |  |  | ✓ |
| Violence Exposure Scale for Children - Revised | I | C |  |  | ✓ |
| Multidimensional Neglectful Behavior Scale | I | C |  |  | ✓ |
| International Physical Activity Prevalence Study Scale | S | PC |  |  | ✓ |
| Questionnaire of Unpredictability in Childhood | S | PC |  |  | ✓ |
| Difficulties in Emotion Regulation Scale | S | PC |  |  | ✓ |
| Behavior Rating Inventory of Executive Function - Adult | S | PC |  |  | ✓ |
| Behavior Rating Inventory of Executive Function – Preschool | S | PC |  |  | ✓ |
| Child’s Sleep Habits Questionnaire | S | PC |  |  | ✓ |
| Everyday Discrimination Scale | S | PC |  |  | ✓ |
| Heightened Violence Scale | S | PC |  |  | ✓ |
| Family Routines Inventory | S | PC |  |  | ✓ |
| Adult Health Questionnaire | S | PC |  |  | ✓ |
| Adult Health Behavior Questionnaire | S | PC |  |  | ✓ |
| Adult ADHD Scale | S | PC |  |  | ✓ |
| Emotion Regulation Checklist | S | PC |  |  | ✓ |
| Child Positive Adjustment Scale | S | PC |  |  | ✓ |
| Social Competence and Self-Esteem Scale | S | PC |  |  | ✓ |
| TV Use and Screen Time Questionnaire (Project Developed) | S | PC |  |  | ✓ |
| Parent Social Class Socialization Measure | S | PC |  |  | ✓ |
| Emotion Reactivity Scale | S | PC |  |  | ✓ |
| Immigration Status Questionnaire (Project Developed) | S | PC |  |  | ✓ |
| Self-Expressiveness in the Family Questionnaire | S | PC |  |  | ✓ |
| Gender Identity Questionnaire | S | PC |  |  | ✓ |

*Note.* T = Task; I = Interview; S = Survey; C = Child; PC = Primary Caregiver.
