# Supplementary material for: Testing a Conceptual Model of Early Adversity, Neural Function, and Psychopathology: Protocol for a Retrospective Observational Cohort Study
Source: JMIR Res Protoc. 2024 Sep 17;13:e59636. doi: 10.2196/59636 (PMC11445632; doi:10.2196/59636)
Supplement: Multimedia Appendix 2 [file resprot_v13i1e59636_app2.pdf]

SUMMARY STATEMENT
(Privileged Communication)

PROGRAM CONTACT:
Julia Zehr
301-443-1617
zehrj@mail.nih.gov

Release Date: 06/29/2017
Revised Date:

Application Number: 1 R01 MH115004-01

Principal Investigator

SHERIDAN, MARGARET ANN

Applicant Organization: UNIV OF NORTH CAROLINA CHAPEL HILL

Review Group: CPDD
Child Psychopathology and Developmental Disabilities Study Section

Meeting Date: 06/22/2017
Council: OCT 2017
Requested Start: 12/01/2017
RFA/PA: PA16-160
PCC: B4-TBX

Project Title: Do dimensions of adversity differentially predict neural development and psychopathology in young children?
SRG Action: Impact Score:37 Percentile:24
Next Steps: Visit https://grants.nih.gov/grants/next_steps.htm
Human Subjects: 44-Human subjects involved - SRG concerns
Animal Subjects: 10-No live vertebrate animals involved for competing appl.
Gender: 1A-Both genders, scientifically acceptable
Minority: 1A-Minorities and non-minorities, scientifically acceptable
Children: 2A-Only Children, scientifically acceptable
Clinical Research - not NIH-defined Phase III Trial

ADMINISTRATIVE BUDGET NOTE: The budget shown is the requested budget and has not been adjusted to reflect any recommendations made by reviewers. If an award is planned, the costs will be calculated by Institute grants management staff based on the recommendations outlined below in the COMMITTEE BUDGET RECOMMENDATIONS section.
EARLY STAGE INVESTIGATOR
NEW INVESTIGATOR

1R01MH115004-01 Sheridan, Margaret

COMMITTEE BUDGET RECOMMENDATIONS
EARLY STAGE INVESTIGATOR
NEW INVESTIGATOR
PROTECTION OF HUMAN SUBJECTS UNACCEPTABLE
SCIENTIFIC REVIEW OFFICER'S NOTES

RESUME AND SUMMARY OF DISCUSSION: The proposed research will examine predictions from the investigators' conceptual model of adversity in which deprivation and threat experiences are related to distinct effects on neural circuitry and to distinct outcomes. The significance of the work lies in a clearer understanding of the mechanisms underlying the effects of early adversity on brain development and the development of psychopathology. Strengths of the application include the strong track record of this new Principal Investigator, a solid research team, a strong scientific premise supported by a clear conceptual framework, the focus on young children, well thought out recruitment procedures, and the emphasis on RDoC constructs of cognitive control and negative valence. Weaknesses include concerns with the operationalization of the threat and deprivation constructs, and the inclusion of extreme exposure groups in the preliminary data but a community sample in the proposed work. Although many saw the translation to younger children to be a significant advance, others noted that the model is already being tested by these investigators in older children, limiting the significance. During discussion, panel members noted that there may be overlap in exposures to threat and deprivation but this was viewed as acceptable as it reflects the real world. Overall, this research tests a novel conceptual model of distinct neural pathways and outcomes related to two types of adversity; although there are some concerns with the measures and some viewed the advance as incremental, the project is likely to have a moderate to high impact on the field of developmental psychopathology.

DESCRIPTION (provided by applicant): Early adversity profoundly affects diverse aspects of child development, including brain development, physiological reactivity to stress, and long-term risk for mental illness. Most models of these effects focus on the number rather than character of adverse childhood experiences. The current proposal tests a novel conceptual model focused on the type of exposure, which differentiates two primary dimensions of experience underlying multiple forms of adversity: deprivation and threat. Deprivation involves a lack of enriching and species expectant cognitive and social inputs (e.g., neglect). Threat involves actual or perceived danger to the physical integrity of the child (e.g., exposure to violence). Here we test the hypothesis that deprivation and threat increase risk for psychopathology through separable neurobiological pathways. We identify these pathways using basic animal and human neuroscience and present them as compliments to existing models. The proposed project will examine the impact of deprivation and threat on the development of neural networks in Cognitive Control Systems and Negative Valance Systems. We predict that early deprivation exposure results in reduced cognitive control, and disruptions in the neural systems supporting cognitive control. Early threat exposure, in contrast, results in disrupted fear learning and alterations in fear circuitry. The current proposal extends our previous work to include longitudinal prediction of psychopathology during middle childhood and in depth evaluation of the proposed neurodevelopmental mechanisms. It is widely hypothesized that adversity primarily influences neural development during early childhood, yet these associations are most often studied in adolescence and adulthood. Here we propose investigating early adversity exposure in young children aged 4-7 years when these systems are undergoing peak development. To assess deprivation and threat in young children we will use in-depth home assessments including observational and parent-report measures. All children will complete structural MRI and functional neuroimaging using EEG and fMRI. The PI has extensive experience collecting such measures from children in this age range and this work follows

directly from her recently completed K01 award. Understanding neurodevelopmental processes linking adversity to psychopathology will open up new pathways to prevention and intervention. The proposed research would directly address Objectives 1 and 2 of the NIMH strategic plan.

PUBLIC HEALTH RELEVANCE: Early adversity profoundly affects short and long term risk for mental illness, along with many other diverse aspects of child development. Identifying neural pathways through which adversity comes to impact mental health is of central importance for improving prevention and intervention of psychopathology following these exposures and addresses Objectives 1 and 2 of the NIMH strategic plan. The current study is designed to identify pathways from early risk to psychopathology considering the impact of developmental environment on neural function/structure.

CRITIQUE 1

Significance: 1
Investigator(s): 2
Innovation: 2
Approach: 3
Environment: 1

Overall Impact: This R01 application seeks to test a model of the Principal Investigator and Co-investigator that adversity can be categorized into specific types of experiences: deprivation and threat. It is expected that these types of adversity will have specific effects on brain development and lead to distinct outcomes. Early deprivation exposure will result in reduced cognitive control and affect RDoC Cognitive Systems; and threat exposure will lead to disrupted fear learning and affect the RDoC Negative Valence System. In addition, in an exploratory aim, the investigators hypothesize that a lack of predictability in the environment (e.g., family instability) will result in reduced reward learning and impact the RDoC Positive Valence System. To accomplish their objectives, the investigative team will collect multi-level data on a sample of children (N = 228) between 4 and 7 years of age. The recruitment strategy will ensure a range of exposures to deprivation and threat and that these experiences are not collinear and so these experiences can be differentiated in the analysis. Moreover, their prior work shows that these experiences can be statistically separated. Data collection includes detailed observational measures of the family at home using video recording and LENA to record and code language interactions. Moreover, parent report measures of the home and neighborhood environments will be acquired and parents will complete a diagnostic interview about the child. In addition, structural MRI, task-based fMRI and EEG and, if time allows, resting connectivity and DTI, will be acquired. There will also be a longitudinal follow-up two years later focusing on the mental health of the child. Support for this theory that examines specific forms of adversity would be significant, because it would identify the particular ways by which adversity gets under the skin and increases the likelihood of various negative outcomes. This knowledge could lead to improved prevention and intervention strategies. Therefore, the significance of the work is high. Moreover, while members of the research team are examining this model in older samples, this is the first test in young children and so the work is innovative. The Principal Investigator is an outstanding new investigator with a highly relevant track record. In addition, she has assembled a very strong research team to perform the work. There are two moderate concerns with the application. First no data sharing plan is offered. While it is not required, freely sharing the data through a server, such as the RDoC DB, would have increased the impact of the work. Therefore, this is a missed opportunity. Second, DTI and resting fMRI procedures (acquisition and analyses) are not clearly described; nor is it clear who has the expertise on the team to ensure data quality. Nevertheless, the DTI and resting fMRI data are less vital to the project and so this concern is reduced somewhat. Overall, considering the strengths and the two modest weaknesses, the work is expected to have a sustained impact on the field.

1. Significance:

Strengths

- If the model is supported, the work would be highly significant. It would identify mechanisms by which adverse environments alter brain development and increase the likelihood of poor outcomes.

Weaknesses

- None noted.

2. Investigator(s):

Strengths

- The Principal Investigator has an impressive track record and she has conducted relevant work to the project.
- The Principal Investigator has assembled a strong team that increases the likelihood that the work well be done.

Weaknesses

- It is not clear who has the expertise to acquire and analyze the DTI and resting fMRI data.

3. Innovation:

Strengths

- This approach to studying adversity has been studied in older samples. This is the first time that the model would be examined in young children.

Weaknesses

- None noted.

4. Approach:

Strengths

- Overall, the approach appears to be sound.

Weaknesses

- DTI and resting fMRI acquisition and analyses are not clearly described. These data are less vital to the project and so this is not a major concern.
- No data sharing plan is provided. By not sharing the data, the potential impact of the work is reduced. In addition, it makes it less likely that problems are discovered.

5. Environment:

Strengths

- The environment is outstanding.

Weaknesses

- No weaknesses were noted.

Protections for Human Subjects:

Acceptable Risks and/or Adequate Protections

- Protections are in place and risks are acceptable.

Data and Safety Monitoring Plan (Applicable for Clinical Trials Only):

Not Applicable (No Clinical Trials)

Inclusion of Women, Minorities and Children:

- Sex/Gender: Distribution justified scientifically
- Race/Ethnicity: Distribution justified scientifically
- For NIH-Defined Phase III trials, Plans for valid design and analysis: Not applicable
- Inclusion/Exclusion of Children under 18: Including ages <18; justified scientifically
- The study will include both sexes, a good representation of races as well as ethnicities and subjects are all children. This is all appropriate and justified.

Vertebrate Animals:

Not Applicable (No Vertebrate Animals)

Biohazards:

Not Applicable (No Biohazards)

Resource Sharing Plans:

Not Applicable (No Relevant Resources)

- A resource sharing plan was not provided. While it may not be required, execution of such a plan would increase the impact of the work.

Authentication of Key Biological and/or Chemical Resources:

Not Applicable (No Relevant Resources)

Budget and Period of Support:

Recommend as Requested

CRITIQUE 2

Significance: 3
Investigator(s): 2
Innovation: 4
Approach: 4
Environment: 1

Overall Impact: The project seeks to examine the nature of exposure to adversity, defined as deprivation and threat, on the neurobiological system of the brain in a sample of youth ages 4-7 years.. One of the main advantages of the current project is the focus on a younger age range than is typically included in neurobiological trauma research. However, it is not clear how different the current application is from ongoing work of the Principal Investigator and co-investigator. Although the specific research questions are different and the current application includes an exploratory aim for predictability – the processes under investigation are similar – how deprivation impacts cognitive functioning and how threat impacts negative valence systems in the brain (and positive valence as exploratory). The current application seeks to test if indeed deprivation and threat experiences are different in the ways the brain is impacted and the investigators suggest several brain systems likely implicated although these are not particularly novel - cognitive control areas of the brain will be associated with deprivation and emotion centers and systems impacted by threat experiences. Although there is likely some overlap in both the cognitive and emotional pathways, the project seeks to determine where the overlap is not and what cortical functions are related to deprivation and which are related to threat. There is a good development for the case for Aim 1. The investigators seek to examine multiple neurobiological systems examined separately in previous research altogether to confirm that these systems are also relevant or active in a younger sample. The preliminary studies supporting Aim 1 are strong – although fairly restricted to ADHD behaviors as outcomes making it less clear how or why other pathology (e.g. internalizing behaviors) are included in the current predictions. The project will also examine these processes over time to establish the stability of the processes documented in Aim 1. Aim 2 is promising but somewhat under-developed in that the application suggests that the neurobiological pathways identified in Aim 1 will operate as mediators between exposure and subsequent internalizing and externalizing disorders but are not specific to the nature of the expected pathology, essentially suggesting that disrupted brain processes are related to almost all forms of later pathology in youth. The lack of specificity in Aim 2 lessens the potential impact of this aim. Aim 3 is exploratory and seeks to add predictability of adverse events and this is a novel idea, but is not well supported by preliminary studies as it is not clear that predictability is a distinct construct or experience from threat or deprivation. The measurement of aspects of adversity are not clearly linked to actual experiences of threat or deprivation. The research team is strong as the Principal Investigator has expertise in the constructs of interest and the rest of the team offer somewhat complementary expertise although the role for each member is not clear. The team has significant experience in large-scale studies and in recruiting high-risk youth and families although work with the current age range is somewhat limited. Impact is likely to be moderate.

1. Significance:

Strengths

- The application will focus on the neurobiological processes underlying exposure to adversity through two well-developed constructs (e.g., cognitive control and negative valence systems).

Weaknesses

- The third process suggested in the application is exploratory - that chaos experiences impact the positive valence system. The potential impact of this aim or discrimination of this construct from other aspects of adversity is not well developed.
- Because threat and deprivation tend to co-occur in children – it is not clear how significant it is to disentangle them from each other – even if there are separate neural pathways that demonstrate their unique effects – in the real world both happen to the same children and at and over the same course of time making the impact of the findings potentially less significant.

2. Investigator(s):

Strengths

- Generally, the research team is strong with Dr. Sheridan as the Principal Investigator as she is a clinical psychologist with a solid history of federal funding as a Co-I on several similar and related projects. She has the expertise to oversee the project to completion.

Weaknesses

- Although the Principal Investigator has been Co-Investigator on several similar projects – it is not clear that she has the experience needed (perhaps even moreso in a new setting) to recruit the kinds of families required for the project. Little information is provided outlining the challenges that are common to this kind of research or remedies for how these problems will be managed.
- Dr. McLaughlin is a Co-Investigator but not included in the budget justification. Dr. Hussong's role is unclear as she is listed as a consultant and as a Co-Investigator in different places in the application. It is also not clear how much assistance Dr. Hussong can provide directly to the project in her role as Director of the Center for Developmental Science as her areas of expertise are not clearly linked with the aims of the proposed project.
- How the investigative team has experience with home visits with families exposed to significant adversity needs clarification in the application. The plan includes interviewing youth about their trauma or child maltreatment and given that this is done in some part in interview format where the participant's name is known to the research team, the protections for how past abuse will be managed and reported are not clear

3. Innovation:

Strengths

- The project includes a novel conceptualization of adversity exposure – threat and deprivation rather than the common and likely oversimplified total number of events that is used in most research. One additional construct is the notion of predictability in adverse events that has promise as a novel construct in adversity conceptualization.

Weaknesses

- Although the conceptualization of adversity is novel, it is the same approach taken in the R01 grant of the project on which Dr. Sheridan is a Co-Investigator (Principal Investigator: McLaughlin) thus dampening enthusiasm for the innovation in the current application.

4. Approach:

Strengths

- The approach includes a well-developed set of neurobiological measures and procedures that likely will meet the study aims. The measures include multi-modal approaches to increase the rigor of the study results
- The recruitment procedures are thoughtful. A strength of the project is the extensive attention to detail in recruitment and the multiple resources accessed by the research team to ensure that the threat and deprivation variables can be captured in a sample in a way that maximizes the external validity of the study.
- The inclusion of teacher report is a strength of the study and increases the potential robustness of the study results.

Weaknesses

- The project may be underpowered – especially the SEM analyses and tests for mediation as they appear to be based on a sample size of 200+ and not the final sample estimates provided in the application of 128 youth and caregivers.
- A major concern is the measurement of adversity – the tools included are proxy measures at best (although there are many of them) of the constructs of interest. The rather oversimplified approach to capturing threat and deprivation is a weakness of the project. For example, the plan is to include measures of bullying and life events and observations of parent-child interaction – none of these are clearly linked to how much “threat” the child has experienced and seem to be more of a mix of measures of life experiences and qualities of the parent-child interaction in a rather artificial setting. The same is true for the operationalization of deprivation that includes measures of language complexity and cognitive stimulation from a free-play paradigm. There is no clear convincing evidence provided that any of these measures of adversity are actually capturing the dose of threat or deprivation or chaos present in the child’s life.
- Although the recruitment plan is interesting, it is not clear how families who come to the hospital due to injury (which the application seems to assume is traumatic) and focusing on the ones from low income areas will be sufficient to recruit the necessary sample. Although other members of her team have experience with the ib2b system, it is not clear how this sample is comparable to the needs of the project.
- The measurement of deprivation is problematic as it appears to be an oversimplification of what it means to experience deprivation beyond financial. Using number of books in the home seems a rather outdated way to capture deprivation in modern times. Part of what is theoretically interesting and innovative is the sample – how youth exposed are different or how youth exposed to different kinds of things are different or how different kinds exposures affect the brain differently - this important question requires a very solid capture of the sample of interest and their experiences and the application is underdeveloped in this aspect.
- It is not clear how the investigators will know what was or was not a threatening experience from the child aside from an a priori list of the events that the investigators assume are threatening experiences. Families from these communities may disagree.

5. Environment:

Strengths

- The University of North Carolina at Chapel Hill has the necessary resources to meet the study aims.

Weaknesses

- None noted.

Protections for Human Subjects:

Unacceptable Risks and/or Inadequate Protections

- More information is needed on the protection for child participants who express past abuse. Human subjects issues are not well explained in the application, especially the assessment of maltreatment. The plan is to include the LENA system and it is not clear from the application that the investigative team understands the challenges and human subject risks associated with using this technology with the intended sample.

Data and Safety Monitoring Plan (Applicable for Clinical Trials Only):

Not Applicable (No Clinical Trials)

Inclusion of Women, Minorities and Children:

- Sex/Gender: Distribution justified scientifically
- Race/Ethnicity: Distribution justified scientifically
- For NIH-Defined Phase III trials, Plans for valid design and analysis: Not applicable
- Inclusion/Exclusion of Children under 18: Including ages <18; justified scientifically
- The project will enroll children ages 4-7 and their caregivers with a reasonable distribution of ethnicity and gender.

Vertebrate Animals:

Not Applicable (No Vertebrate Animals)

Biohazards:

Not Applicable (No Biohazards)

Budget and Period of Support:

Recommend as Requested

CRITIQUE 3

Significance: 4
Investigator(s): 2
Innovation: 3
Approach: 5
Environment: 2

Overall Impact: This application addresses an important issue by attempting to distinguish the neurobiological pathways associated with different types of early-life adversity, specifically deprivation, threat and low predictability, in determining the risk for psychopathology. Such information can be helpful in developing more personalized interventions for the three subgroups. The scientific premise is based on a well-grounded theoretical model but the preliminary data linking prediction of these different types of adverse experiences to the hypothesized neural circuits are not strong and are based on extreme levels of deprivation and threat when the proposed research focuses on a general community sample with high levels of poverty but without a clear delineation of inclusion/exclusion criteria to distinguish the different groups. The study is conceptually novel in considering the alternate pathways of cognitive control, negative valance and reward systems in these different types of adversity although the methods employed to assess these systems are not novel. The research team is well-positioned to conduct the study with complementary, inter-disciplinary skills necessary for the execution of the protocol. Despite these strengths, the study will likely have only moderate impact because the feasibility of identifying discrete groups with unique adversity experiences and how well these reflect naturally occurring groups is not clear. The proposed proxy for deprivation, namely low parental education, does not encompass other types of deprivation commonly seen in families with trauma. Overall, the impact is likely to be moderate.

Protections for Human Subjects:

Acceptable Risks and/or Adequate Protections

- The risks were well-delineated, and appropriate measures were instituted to minimize risks.

Data and Safety Monitoring Plan (Applicable for Clinical Trials Only):

Not Applicable (No Clinical Trials)

Inclusion of Women, Minorities and Children:

- Sex/Gender: Distribution justified scientifically
- Race/Ethnicity: Distribution justified scientifically
- For NIH-Defined Phase III trials, Plans for valid design and analysis: Not applicable
- Inclusion/Exclusion of Children under 18: Including ages <18; justified scientifically
- Study includes a narrow age group (4-7 years) but this is scientifically justified.

Vertebrate Animals:

Not Applicable (No Vertebrate Animals)

Biohazards:

Not Applicable (No Biohazards)

Resource Sharing Plans:

Unacceptable

- No resource sharing plan is provided. If the study achieves the aims, these data can be useful for the larger research community.

Authentication of Key Biological and/or Chemical Resources:

Not Applicable (No Relevant Resources)

Budget and Period of Support:

Recommended budget modifications or possible overlap identified:

- In the application, the Principal Investigator describes her expertise in developmental psychopathology, neuroimaging and samples with childhood adversity. It is not clear how Dr. McLaughlin's expertise will add to this, particularly from a distance. She is budgeted for 20% time in Years 1-5. This is a high percentage of effort without clear delineation of her expertise from that of the Principal Investigator.

THE FOLLOWING SECTIONS WERE PREPARED BY THE SCIENTIFIC REVIEW OFFICER TO SUMMARIZE THE OUTCOME OF DISCUSSIONS OF THE REVIEW COMMITTEE, OR REVIEWERS' WRITTEN CRITIQUES, ON THE FOLLOWING ISSUES:

PROTECTION OF HUMAN SUBJECTS: UNACCEPTABLE

Most risks and protections are adequately addressed. However, there are concerns with the collection of data in the home and with the potential for reports of past abuse.

INCLUSION OF WOMEN PLAN: ACCEPTABLE
Females are included in the study design.

INCLUSION OF MINORITIES PLAN: ACCEPTABLE
Minorities are included in the study design.

INCLUSION OF CHILDREN PLAN: ACCEPTABLE
Children, 4 to 7 years of age, are the focus of the proposed research.

COMMITTEE BUDGET RECOMMENDATIONS: Cl**earer justification for the proposed effort of co-investigator McLaughlin is needed given the overlap in expertise with the Principal Investigator.**

SCIENTIFIC REVIEW OFFICER'S NOTES: The NIH special practice for new investigator R01 applications reviewed in the Center for Scientific Review study sections applies to this application. Resubmission (amended -A1) R01 applications from new investigators may be submitted on a special receipt date for review in the very next review cycle. See this notice in the NIH Guide for Grants and Contracts for more details: http://grants.nih.gov/grants/guide/notice-files/NOT-OD-11-057.html.

You should contact the NIH program officer whose name is shown in the upper left hand corner of page one of this Summary Statement for information about whether this application may be fundable or whether you will need to submit an amended application. The program officer can also help you decide whether the changes and improvements necessary to address the weaknesses noted in the reviewers' critiques could be accomplished in the relatively short time available. You are also strongly advised to seek input from mentors, your Department chair, etc.

If you choose to submit a resubmission application for the next review cycle under this policy for new investigators, your amended application must be received at NIH no later than Thursday, August 10, 2017.

You may, of course, choose to take more time to resubmit your application. If so, you should prepare the resubmission for the normal dates for amended applications as specified in this table: http://grants1.nih.gov/grants/funding/submissionschedule.htm.

Footnotes for 1 R01 MH115004-01; PI Name: Sheridan, Margaret Ann

NIH has modified its policy regarding the receipt of resubmissions (amended applications). See Guide Notice NOT-OD-14-074 at http://grants.nih.gov/grants/guide/notice-files/NOT-OD-14-074.html. The impact/priority score is calculated after discussion of an application by averaging the overall scores (1-9) given by all voting reviewers on the committee and multiplying by 10. The criterion scores are submitted prior to the meeting by the individual reviewers assigned to an application, and are not discussed specifically at the review meeting or calculated into the overall impact score. Some applications also receive a percentile ranking. For details on the review process, see http://grants.nih.gov/grants/peer_review_process.htm#scoring.
