# Supplementary figures and images for: Testing a Conceptual Model of Early Adversity, Neural Function, and Psychopathology: Protocol for a Retrospective Observational Cohort Study
Source: JMIR Res Protoc. 2024 Sep 17;13:e59636. doi: 10.2196/59636 (PMC11445632; doi:10.2196/59636)

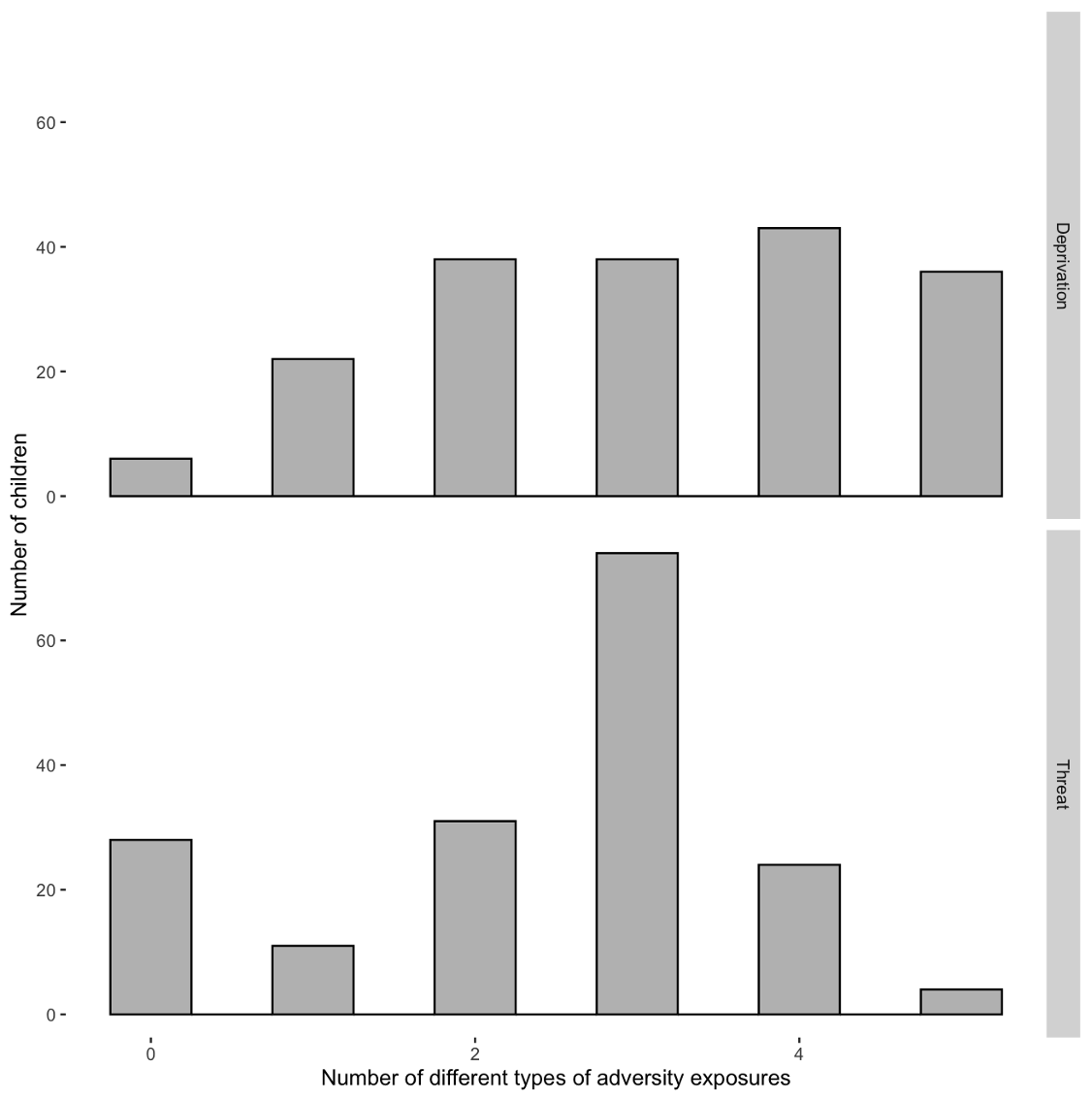

Supplement: Multimedia Appendix 3 [file resprot_v13i1e59636_app3.png]
